# Supplementary material for: Comprehensive investigation and regulatory function of lncRNAs engaged in western honey bee larval immune response to Ascosphaera apis invasion
Source: Front Physiol. 2022 Dec 16;13:1082522. doi: 10.3389/fphys.2022.1082522 (PMC9800914; doi:10.3389/fphys.2022.1082522)
Supplement: Supplementary file 1 [file Table1.DOCX]

**SUPPLEMENTARY TABLE 1** | Detailed information of primers used in this work

| Name | Sequence (5’-3’) |
| --- | --- |
| MSTRG.1133.1-F | ACGAGGAGGAGGGTGGTGAAGA |
| MSTRG.1133.1-R | GAGCACACACGCACACGGAAA |
| XR_001704875.2-F | CCTGGCCGTTACTTCCGTGGTA |
| XR_001704875.2-R | GGCGATTGTTGGCGGTATCTCT |
| MSTRG.11613.1-F | GCGTGTGCATCCGAGCGATT |
| MSTRG.11613.1-R | CGACGGAGAGCGAAGGAAGAGA |
| MSTRG.4918.2-F | GTGGTGGAGGTGGTTGGTGGTA |
| MSTRG.4918.2-R | ACTGGTGGTGATCGGTGAGCAA |
| MSTRG.9603.5-F | TGGCAGTAGACCGTTCGCAAGA |
| MSTRG.9603.5-R | TCGCTTGACCTCGTCGCTGAA |
| MSTRG.9603.5-F | TGGCAGTAGACCGTTCGCAAGA |
| MSTRG.9603.5-R | TCGCTTGACCTCGTCGCTGAA |
| XR_001705688.2-F | GGTGAGTGATCCGAGTCAGTGA |
| XR_001705688.2-R | GCAACAAGACACAAGCGACAGT |
| XR_410074.3-F | ACGGTGCCGATATTCGCAAGTT |
| XR_410074.3-R | GCAGACAGCGTCTCTGATGGAA |
| XR_410074.3-F | ACGGTGCCGATATTCGCAAGTT |
| XR_410074.3-R | GCAGACAGCGTCTCTGATGGAA |
| *actin*-F | CACTCCTGCTATGTATGTCGC |
| *actin*-R | GGCAAAGCGTATCCTTCA |

**SUPPLEMENTARY TABLE 2** | Overview of data from stand-specific library-based RNA-seq

| Group | Raw reads | Clean reads | Q20 (%) | Q30 (%) | Mapping ratio (%) |
| --- | --- | --- | --- | --- | --- |
| AmCK1 | 85,811,046 | 85,739,414 | 12,584,612,986 (98.10) | 12,111,701,466 (94.42) | 99.92 |
| AmCK2 | 81,962,296 | 81,896,402 | 12,019,257,367 (98.11) | 11,559,735,865 (94.36) | 99.92 |
| AmCK3 | 85,636,572 | 85,573,798 | 12,019,257,367 (98.11) | 11,559,735,865 (94.36) | 99.93 |
| AmT1 | 79,267,686 | 79,202,304 | 11,624,829,303 (98.07) | 11,177,847,810 (94.30) | 99.92 |
| AmT2 | 82,889,882 | 82,828,926 | 12,175,645,045 (98.25) | 11,736,056,501 (94.70) | 99.93 |
| AmT3 | 100,211,796 | 100,128,692 | 14,710,798,291 (98.16) | 14,159,475,654 (94.48) | 99.92 |

**SUPPLEMENTARY TABLE 3** | Statistics of mapping of clean reads to reference genome

| Group | Genome (%) | Exon (%) | Intron (%) | Intergenic region (%) |
| --- | --- | --- | --- | --- |
| AmCK1 | 43,299,906(92.06) | 27,320,128 (63.10) | 4,151,371 (9.59) | 11,828,407 (27.32) |
| AmCK2 | 62,159,741(94.74) | 43,055,656 (69.27) | 5,317,946 (8.56) | 13,786,139 (22.18) |
| AmCK3 | 67,639,608 (94.20) | 46,129,515 (68.20) | 6,035,301 (8.92) | 15,474,792 (22.88) |
| AmT1 | 58,614,260 (93.69) | 38,810,540 (66.21) | 5,384,610 (9.19) | 14,419,110 (24.60) |
| AmT2 | 61,342,862(94.35) | 41,899,579 (68.30) | 5,269,266 (8.59) | 14,174,017 (23.11) |
| AmT3 | 53,355,422(94.81) | 37,240,371 (69.80) | 4,922,453 (9.23) | 11,192,598 (20.98) |
